# RETRACTED ARTICLE: CKS2 induces autophagy-mediated glutathione metabolic reprogramming to facilitate ferroptosis resistance in colon cancer

**DOI:** 10.1186/s10020-024-00979-5

**Published:** 2024-11-15

**Authors:** Leilei Yang, Chengfeng Fang, Jiaju Han, Yufeng Ren, Zaiping Yang, Lingyan Shen, Dinghai Luo, Ruili Zhang, Yan Chen, Shenkang Zhou

**Affiliations:** 1 Department of Gastrointestinal Surgery, Taizhou Hospital, Wenzhou Medical University, No.105 Westgate Street, Linhai, 317000 China; 2 Key Laboratory of Minimally Invasive Techniques & Rapid Rehabilitation of Digestive System Tumor of Zhejiang Province, Linhai, 317000 China; 3 Department of Anaesthesia, Taizhou Hospital, Wenzhou Medical University, Linhai, 317000 China; 4 Department of Gastroenterology, Taizhou Hospital, Wenzhou Medical University, Linhai, 317000 China; 5 Department of Family-oriented wards, Taizhou Hospital, Wenzhou Medical University, No.105 Westgate Street, Linhai, 317000 China

**Keywords:** Cyclin-dependent kinase subunit 2, Autophagy, Ferroptosis, Colon cancer, Glutathione metabolism

## Abstract

**Background:**

Ferroptosis, a form of cell death characterized by lipid peroxidation, plays a crucial role in tumor suppression, offering novel avenues for cancer therapy. Previous studies have indicated that high levels of cyclin-dependent kinase subunit 2 (CKS2) promote the progression of various cancers. However, the potential interplay between CKS2 and ferroptosis in colon cancer (CC) remains unclear.

**Methods:**

Bioinformatics and RNA-seq analyses were employed to study genes associated with the ferroptosis signaling pathway. CKS2 expression was evaluated using quantitative reverse transcription polymerase chain reaction (qRT-PCR) and Western blot (WB). The in vitro and in vivo effects of CKS2 on CC cells were assessed through the CCK-8 assay, colony formation assay, propidium iodide (PI) staining, BODIPY staining, DCFH-DA staining, and animal experiments. Additionally, the impact of CKS2 on autophagy and glutathione (GSH) metabolism was investigated using a transmission electron microscope (TEM), immunofluorescence (IF) assays, WB experiments, and relevant assay kits.

**Results:**

CKS2 expression was elevated in CC, indicating a poor clinical outcome. Knockdown of CKS2 significantly enhanced Erastin-induced ferroptosis in CC cells, leading to reduced GSH metabolism. Conversely, CKS2 overexpression produced opposite effects. Mechanistically, CKS2-induced autophagy reinforced GSH metabolism, thereby increasing resistance to ferroptosis in CC cells. Furthermore, inhibiting CKS2 promoted tumor ferroptosis by downregulating GPX4 expression. Additionally, CKS2 knockdown effectively increased sorafenib-induced ferroptosis both in vitro and in vivo.

**Conclusion:**

CKS2 suppresses ferroptosis in CC by modulating GSH metabolism in both in vitro and in vivo settings. These findings offer new insights into targeting CKS2 for CC treatment and shed light on the mechanism of ferroptosis in CC.

## Introduction

Colon cancer (CC) is one of the most common malignant tumors worldwide (Sung et al. 2021). According to the global cancer statistics in 2020, the incidence and mortality of CC rank fifth among 36 types of cancer, with significant increases in both incidence and mortality rates compared to previous years (Sung et al. 2021). Although continuous progress of diagnostic and treatment methods for colonoscopies such as colon resection, chemotherapy, and immunotherapy have been achieved, the prognosis for advanced CC patients remains unfavorable, with a grim survival rate of 8–30%^2, 3^. Hence, a thorough exploration of the mechanisms behind CC progression is essential to develop innovative therapeutic drugs and enhance the quality of life for patients.

Ferroptosis is a novel form of cell death caused by the accumulation of iron-dependent lipid peroxidation. In ferroptosis, there is an increase in reactive oxygen species (ROS) production, a decrease in mitochondrial volume, and an increase in membrane density (Tang et al. 2021; Jiang et al. 2021). Inhibition of the cystine/glutamate transporter (SCL7A11 or xCT, also known as system Xc-) and glutathione peroxidase 4 (GPX4) is the most common method to induce ferroptosis (Jiang et al. 2021). Dysregulation of ferroptosis is tightly linked with human cancer (Chen et al. 2021). Increasing evidence suggests that the ferroptosis pathway can hinder tumor growth and kill tumor cells (Han et al. 2022; Chen et al. 2023). The interaction between ferroptosis and autophagy is gaining significant attention. Autophagy can selectively break down specific cellular components, supplying materials for ferroptosis, ultimately leading to anti-tumor effects (Zeng et al. 2023). However, ferroptosis is only triggered when autophagy reaches a certain level of intensity (Li et al. 2021). Simultaneously, with ongoing research into how ferroptosis impacts tumors, various cancer treatment strategies centered on enhancing ferroptosis are emerging as focal points of current research. For example, the combination treatment of CC cells with ferroptosis inducer Erastin and cisplatin exhibits the expected sensitization effect (Guo et al. 2018). The deubiquitinase inhibitor PR-619 provoking CC ferroptosis can greatly dampen tumor growth in mice when combined with anti-PD1 therapy (Wu et al. 2023). Hence, focusing on cell ferroptosis could be advantageous in developing more effective anticancer treatments. Nevertheless, the regulatory mechanisms of ferroptosis during CC progression remain largely unknown.

Cyclin-dependent kinase subunit 2 (CKS2) belongs to the cyclin-dependent kinase (CDK) binding protein family, participating in early embryonic development, somatic cell division, and cell cycle progression (Liberal et al. 2012; Martinsson-Ahlzen et al. 2008). These findings suggest that CKS2 may play a functional role in tumorigenesis. Recently, CKS2 has been observed to exert oncogenic influence, showing high expression in various malignant tumors such as esophageal cancer (Zhang et al. 2020), cervical cancer (CC) (Qin et al. 2022), and non-small cell lung cancer (NSCLC) (Wan et al. 2022). In colorectal cancer (CRC), CKS2 enhances cancer cell proliferation by activating the expression of claudin 1^18^. Furthermore, in lung adenocarcinoma (LUAD), RPA3 may repress cell autophagy and reinforce cancer cell proliferation by upregulating CKS2 to activate the AKT/mTOR signaling pathway (Chen et al. 2022a, b). Although CKS2 has been characterized as an oncogene in many human cancers, its role in CC is not clear and its potential mechanism is mainly unexplored.

In this study, we analyzed the expression of CKS2 in CC and elucidated its biological role in CC cells. The study found that CKS2 was upregulated in CC. The correlation between CKS2 and poor prognosis of CC patients was revealed by bioinformatics prediction. High expression of CKS2 suppressed ferroptosis in CC cells. Mechanistically, CKS2 mediated glutathione (GSH) metabolism reprogramming in CC cells by inducing autophagy. Further analysis revealed that the inhibition of CKS2 promoted ferroptosis in CC cells by downregulating GPX4 expression. Additionally, we demonstrated that suppressing CKS2 expression, both in vivo and in vitro, augmented sorafenib (sora)-induced ferroptosis in CC cells. In summary, this study confirmed that CKS2 can function as a negative regulator of ferroptosis, offering a potential therapeutic approach to enhance sora-induced ferroptosis in tumors by targeting CKS2.

## Materials and methods

### Patients and samples

Samples were collected from 10 cases of CC tissues (International Classification of Diseases, 11th edition, code 2B90) and adjacent normal tissues from patients who underwent surgical resection at Taizhou Hospital, Wenzhou Medical University from January 2019 to December 2022. Patients who received neoadjuvant chemotherapy or radiotherapy, as well as those with other cancers, were excluded from the study. Samples were frozen in liquid nitrogen and stored at **−**80 ℃ until use. Before this experiment, written informed consent was obtained from individuals. The experiments were reviewed and certified by the Ethics Committee of Taizhou Hospital, Wenzhou Medical University (approval number: KL20240457).

### Bioinformatics analysis

Gene expression levels in CC samples were obtained from The Cancer Genome Atlas (TCGA) database (normal_count: 41, tumor_count: 480). Differential analysis on differentially expressed genes (DEGs) was carried out by using open-source software based on R. The gene sets showing the biological process (BP) of Gene Ontology (GO) and Kyoto Encyclopedia of Genes and Genomes (KEGG) were downloaded from the gene set enrichment analysis (GSEA) official website and subjected to functional analysis by using the R package. The Gene Multiple Association Network Integration Algorithm (GeneMANIA) online database was employed to predict the correlation between the target gene CKS2 and the inhibitory genes of ferroptosis.

### RNA-seq analysis

Total RNA was extracted from CC cell samples by using TRIzol reagent (Invitrogen, USA). Subsequently, molecular biology-related equipment was applied to assess the purity, concentration, and integrity of the RNA samples to ensure the use of qualified samples for transcriptome sequencing. When the samples passed the quality control, library construction was launched. After library construction was completed, an initial quantification was carried out. The library was diluted. The fragment distribution was detected using the Agilent 2100 DNA 1000 kit. After passing quality control, the accurate quantification of the library effective concentration (concentration > 2nM) was achieved by quantitative reverse transcription polymerase chain reaction (qRT-PCR) to ensure library quality. Qualified libraries were sequenced on the Illumina platform after pooling as required.

### Cell cultivation

Human normal colonic epithelial cell line HIEC-6 and human CC cell lines HT29, LoVo, and HCT116 were sourced from MEISEN CELL (China). HT29 and HCT116 were cultivated in DMEM. HIEC-6 was cultured in DMEM/F12 medium with 10 ng/mL EGF. LoVo was kept in F12K medium. All media were supplemented with 10% fetal bovine serum (FBS) and 1% double antibodies and were placed in a sterile culture box at a constant temperature with 5% CO_2_.

For the induction and inhibition of autophagy, 25 µmol/L rapamycin (Rapa) and 50 µmol/L chloroquine (CQ) were introduced to the corresponding culture media (Ma et al. 2015; Liu et al. 2019). For the inhibition and induction of ferroptosis, 2 µmol/L Ferrostatin-1 (Fer-1) and the Erastin with corresponding IC_50_ concentration were added to the respective culture media (Zheng et al. 2022; Ye et al. 2021). The rescue experiments were performed by adding recombinant GPX4 protein (EIAab, China) solution into the culture system. Rapa, CQ, Fer-1, and Erastin were all purchased from MCE (USA).

## Cell transfection

We harvested cells during the logarithmic growth phase, seeded them, and replaced the standard culture medium 30 min before reaching approximately 70% confluence. Plasmids containing short hairpin RNA (sh-NC/sh-CKS2) and overexpression gene (oe-NC/oe-CKS2) were obtained from Fenghui Biotechnology (China). We transfect sh-NC/sh-CKS2 and oe-NC/oe-CKS2 plasmids into cells with the use of Ultra Fection 3.0. After incubating in a saturated humidity culture chamber at 37 ℃, 5% CO_2_ for 6 h, the culture medium was replaced with a fresh one for continued cultivation.

### qRT-PCR

Total RNA was extracted from tumor tissues or cells using TRIzol (CWBIO, China). The extracted RNA was reverse transcribed into cDNA using Hifair^®^ II 1st Strand cDNA Synthesis Kit (YESEN, China). qRT-PCR was performed by using uGreener Flex qPCR 2XMix (U&GBIO, China) on an ABI7500 real-time fluorescence quantitative PCR system (Applied Biosystems, USA). GAPDH was used as an internal control, and the calculation method was 2^−ΔΔCt^. The primer sequences are outlined in Table 1.

**Table 1:** Primer set for qRT-PCR

Gene	Primer sequence (5′→3′)
CKS2	F: CGAGTACCGGCATGTTATGT
	R: AAGTCTCCTCCACTCCTCTT
GPX4	F: CGATACGCTGAGTGTGGTTT
	R: CGGCGAACTCTTTGATCTCTT
SLC7A11	F: GTGCTCCTGGTTCTGTTCTT
	R: CTCGGGTGTCTTGTCACTTT
GAPDH	F: GAAGGTCGGAGTCAACGGAT
	R: CCTGGAAGATGGTGATGGGAT

### Western blot (WB)

The cells were lysed in lysis buffer (containing 1% phosphatase inhibitor and 1% protease inhibitor) on ice for 10 min at 4 ℃, 12,000 rpm. The supernatant was gathered after being centrifuged for 15 min and the total protein content was determined using the bicinchoninic acid (BCA) assay kit (Solarbio, China). Denatured proteins were separated by SDS-PAGE, transferred to the PVDF membrane, blocked with 5% skim milk, and then mixed with CKS2 (ABclonal A3791, China), LC3B (LC3I, LC3II ABclonal, China, A5618), p62 (ABclonal, China, A19700), GPX4 (AbclonalA11243, China), and SLC7A11 (AbclonalA13685, China) for overnight incubation at 4 ℃. The TBST washing lasted 3 times, 5 min for each time. Then the membrane was incubated with the secondary antibody (Beyotime A0208, China) at room temperature for 1.5 h. Following three rounds of TBST washing, lasting five minutes each, the protein bands were visualized using the ECL detection kit (Biosharp, China) and photographed with the ChemiScope 6200 imaging system (Clinx, China).

### CCK-8 assay

Transfected CC cells were plated in a 96-well plate (density of 2000 cells/well), with cell viability detected at 0, 24, 48, and 72 h. 10 µl of CCK-8 reagent was introduced to each well, followed by 2 h of incubation at 37 ℃, with the absorbance optical density (OD) value of each well at 450 nm measured by a microplate reader.

We seeded 2 × 10^4^ cells in a 96-well plate and treated them with different concentrations of ferroptosis inducer Erastin (0/5/10/20/40/80 µM) or sora (0/2.5/5/10/20 µM). After 24 h, each well was supplemented with 10 µl of CCK-8 solution and kept at 37 ℃ for 2 h. OD and IC_50_ values were detected and calculated.

### Colony formation assay

Cells were inoculated into a 12-well plate (200 cells/well) and cultivated for 7 days. Then, the plate was immobilized with 75% methanol, stained with 1% crystal violet for 30 min, rinsed with phosphate-buffered saline (PBS), dried at 37 ℃, and photographed with a Nikon D5600 camera (Nikon, Japan) to record the cell units in each well.

#### Transmission electron microscope (TEM)

Cells were inoculated into a T75 culture bottle and transfected with sh-NC/sh-CKS2 and oe-NC/oe-CKS2 plasmids respectively. When the cell density reached 90% or more, cells were gathered by trypsin digestion and washed with PBS, with cell precipitate fixed in 2.5% glutaraldehyde solution. Subsequently, the fixed cells were subjected to permeabilization and embedded overnight at 70 ℃. Embedded slices were stained with 1% uranyl acetate and 0.4% lead citrate, and then observed under the TEM.

### Measurement of ROS and lipid ROS in cells

With the use of the ROS detection kit (Beyotime, Cat. No.: S0033S, China), the level of ROS in cells was determined. Subsequently, cells in each treatment group were seeded in 6-well plates and incubated for 12 h. We diluted the original DCFH-DA solution in the kit with the serum-free medium at a ratio of 1:1000, then added it to each well and cultivated it at 37 ℃ for 30 min. Cells were collected and rinsed three times with PBS solution to remove residual DCFH-DA dye. On an Agilent NovoCyte flow cytometer (Agilent, USA), we finished the analysis.

Next, cells or tumor tissue homogenate were resuspended in 500 µL PBS solution containing 2 µM BODIPY581/591 C11 (MCE, USA), and housed in a CO_2_ incubator at 37 ℃ for 30 min. Samples were analyzed using the Agilent NovoCyte (Agilent, USA).

### Propidium iodide (PI) staining

The Hoechst 33,342/PI dual staining kit (Solarbio, Cat. No: CA1120, China) was employed to assess apoptosis. Cells from each treatment group were inoculated into a 6-well plate and maintained for 12 h. Subsequently, the cells were collected, and to each tube, 5 µL of PI reagent and 5 µL of Hoechst reagent were added. The tubes were then incubated on ice for 20 min and the analysis was performed using the Agilent NovoCyte flow cytometer (Agilent, USA).

### Detection of GSH, glutamate, cysteine, and cystine intake levels and GSH/ oxidized GSH (GSSG) ratio

Relative GSH levels and GSH/GSSG ratios in cells or nude mouse tumor tissues were determined using GSH and GSSG detection kits (Beyotime, Cat. No.: S0053, China), with OD measured at 405 nm. The relative GSH level and GSH/GSSG ratio in the sample were calculated.

The glutamate colorimetric assay kit (Elabscience, Cat. No: E-BC-K903-M, China) was employed to determine glutamate concentration in cells or nude mouse tumor tissues, with OD measured at 450 nm. The glutamate concentration in samples was calculated.

Cysteine concentration in cells or nude mouse tumor tissues was assessed using a cysteine colorimetric test kit (Elabscience, Cat. No.: E-BC-K352-M, China), with OD measured at 600 nm and cysteine concentration calculated. The cystine uptake fluorescence assay kit (Elabscience, Cat. No: E-BC-F066, China) was applied in the measurement of cystine uptake level. The fluorescence values were measured using a SynergyH1 fluorescence microplate reader (Boteng, USA) at an excitation wavelength of 485 nm and an emission wavelength of 535 nm, with the cystine uptake in the samples calculated.

### Immunofluorescence (IF)

Cells were immobilized in 75% alcohol for 30 min, followed by three times rinses with PBS (5 min for each), 10 min of permeabilization with 0.1% TritonX-100, as well as 1 h of block with 5% BSA at room temperature. The slides were incubated overnight at 4 ℃ with LC3B antibody (ABclonal, China, A5618) and then with the secondary antibody conjugated with AF555 (Bioss, China, bs-0295G-AF555) at room temperature for 1 h. After the addition of DAPI (10 ug/ml) (Solarbio, Cat. No: C0065, China) for nuclear staining, the slides were incubated for 5 min, washed with PBS, sealed, and observed under an inverted fluorescence microscope.

### Immunohistochemistry (IHC)

4 µm-thick of CC tumor tissue sections were applied. In brief, we embedded the tissue in paraffin, then deparaffinized the sections and rehydrated them through graded alcohols, followed by 3 times washing in PBS, each for 5 min. Next, the sections were incubated overnight with primary antibodies GPX4 (A11243, abclonal, China) and SLC7A11 (A13685, abclonal, China). The sections were then incubated with secondary antibody goat anti-rabbit IgG at 37 ℃ for 30 min, stained with a working solution of 3, 3′-DAB for 3 min, and rinsed 3 times in PBS, each time for 5 min. Finally, the sections were counterstained with hematoxylin and sealed for microscopic examination. Three sections were taken from each tumor tissue for testing. Five independent fields were randomly selected for observation and photography by using a Leica microscope (×200).

### Xenograft model

Twenty-four female BALB/c nude mice (4 weeks old, 18.0 ± 2.0 g) purchased from Shanghai SLAC Laboratory Animal Co., Ltd. were randomly divided into two groups for in vivo xenograft experiments. The mice were injected with 5 × 10^6^ CC cells with stable knockdown of CKS2 (sh-CKS2) or control vector (sh-NC). The cells were subcutaneously injected into the right axilla of the mice in both groups. When the tumor volume reached 100 mm (Yuan et al. 2021), the nude mice were intraperitoneally injected with sora (5 mg/kg) or PBS. Tumor size was measured every 5 days, and tumor volume was calculated as follows: tumor volume = (length × width^2^)/2. All animal experiments and procedures were guided and approved by Taizhou Hospital, Wenzhou Medical University Ethics Committee (approval number: tzy-2024084).

### Data analysis

Three parallel groups were set up for each group, and the experiment was independently repeated three times. Data analysis was processed using SPSS 22.0 software. Quantitative data were expressed as mean ± standard deviation (*x̅*±s). Independent sample *t*-test was employed for comparison between two groups, while the one-way analysis of variance (ANOVA) was employed for comparison among multiple groups. A *p*-value < 0.05 referred to a significant difference. Statistical charts were drawn by utilizing GraphPad Prism 8.0 software.

## Experimental results

### Expression of CKS2 in CC tissues and cells

**Fig. 1:**
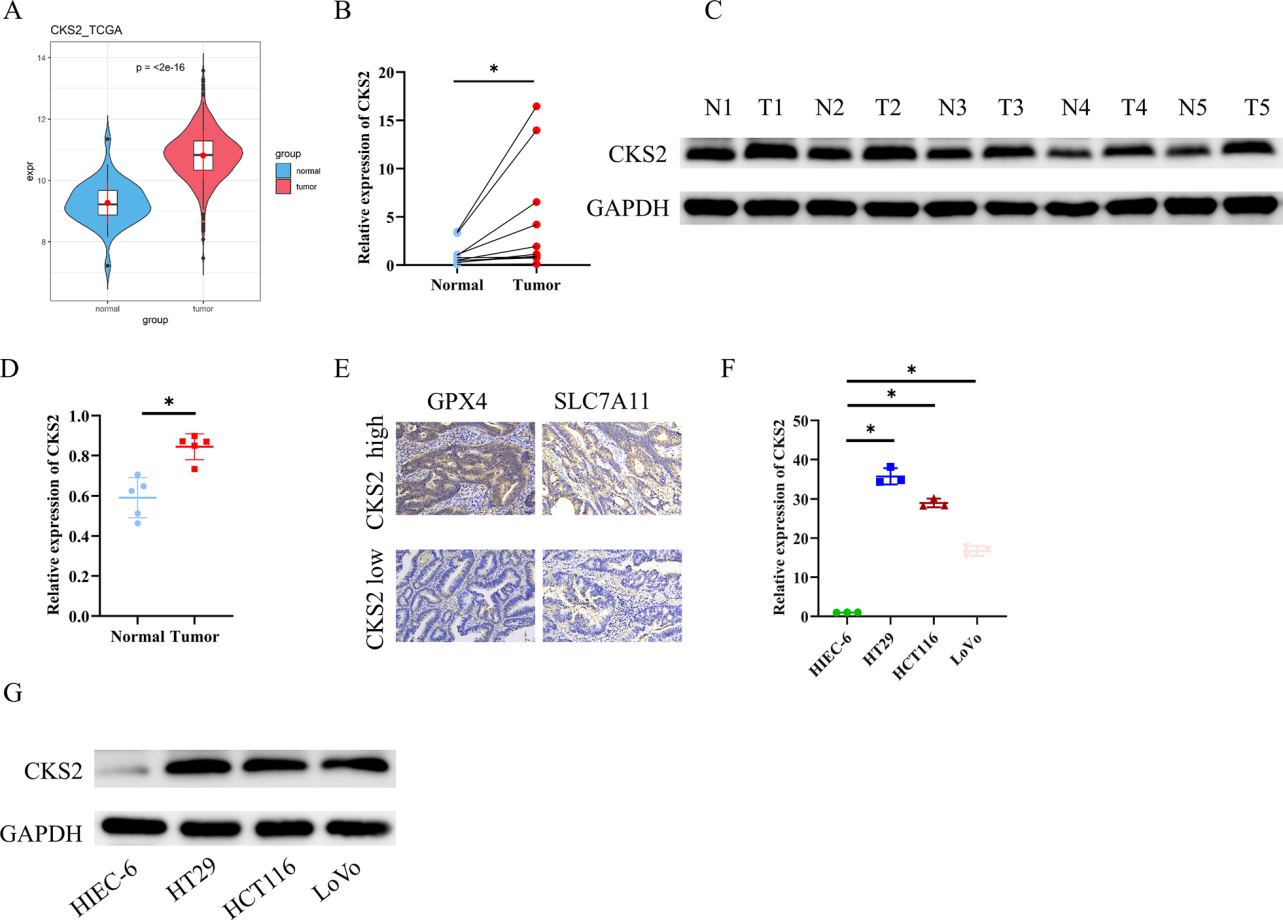
Expression of CKS2 in CC tissues and cells **A** Analysis of CKS2 expression in human normal colon tissues (*n* = 41) and human CC tissues (*n* = 480) using the TCGA database; **B–D** qRT-PCR and WB analysis of CKS2 expression in normal tissues (*n* = 10) and tumor tissues (*n* = 10) of CC patients; **E** IHC evaluation of GPX4 and SLC7A11 levels in patients with different CKS2 expression (*n* = 2); **F–G** qRT-PCR and WB analysis of CKS2 expression in human CC cell lines (*n* = 3). Data are presented as the mean ± SD of three independent experiments. Statistical analysis was conducted by t-test. * means *P* < 0.05

To investigate the potential role of CKS2 in CC, we analyzed the expression of CKS2 using the TCGA database. The analysis revealed that the expression of CKS2 was significantly higher in tumor tissues compared to normal tissues (Fig. 1A). Furthermore, this result was validated in clinical samples through qRT-PCR and WB experiments (Fig. 1B–D). Furthermore, IHC results confirmed that the levels of GPX4 and SLC7A11 were significantly elevated in patients with high CKS2 expression compared to those with low CKS2 expression (Fig. 1E). qRT-PCR and WB results demonstrated that both mRNA and protein levels of CKS2 in CC cell lines HT29, HCT116, and LoVo were significantly elevated than those in normal colonic epithelial cell line HIEC-6 (Fig. 1F and G). Among them, the expression of CKS2 was low in LoVo cells and high in HT29 cells. Therefore, the two cell lines were selected for subsequent experiments. Taken together, CKS2 is significantly upregulated in CC tissues and cells.

### The effect of CKS2 on ferroptosis of CC cells

**Fig. 2:**
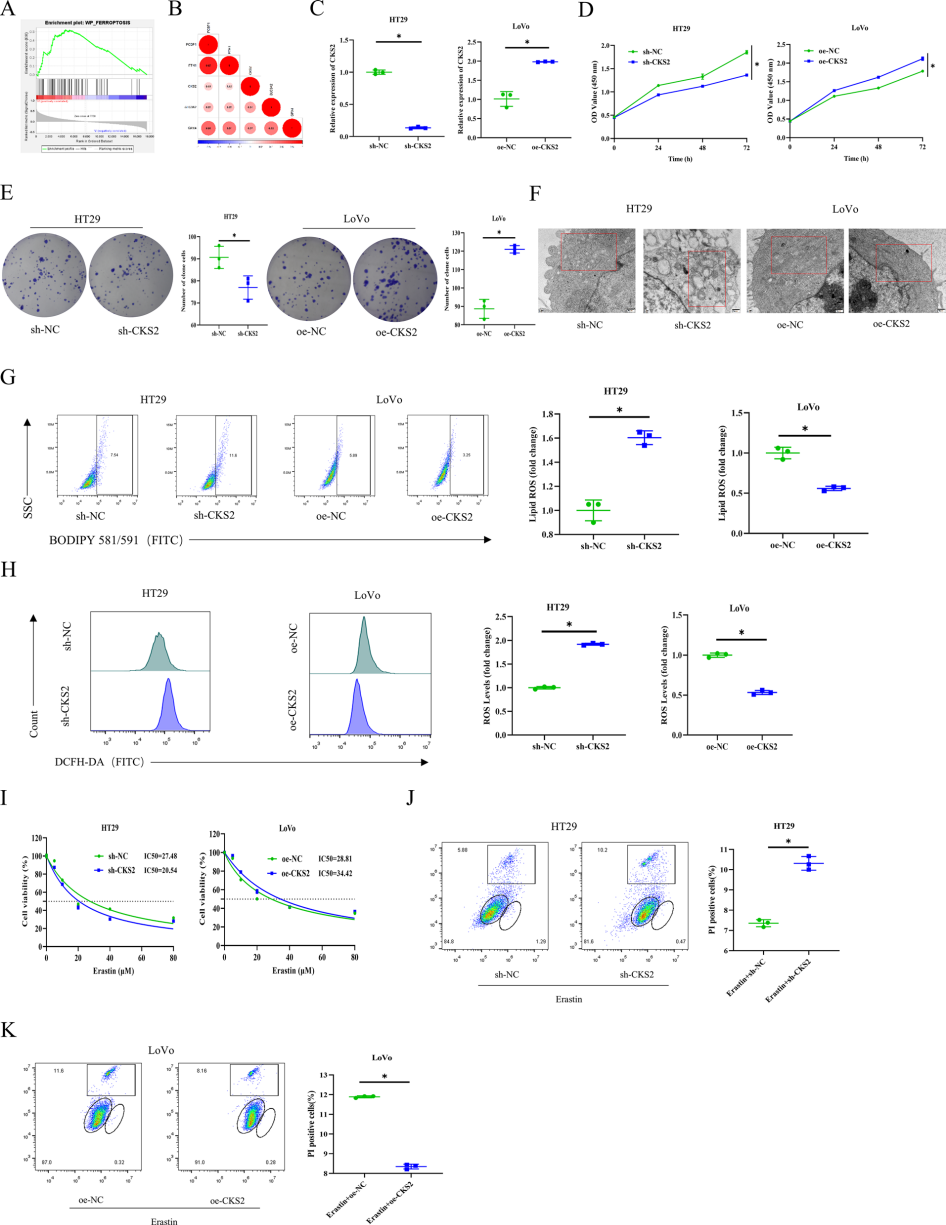

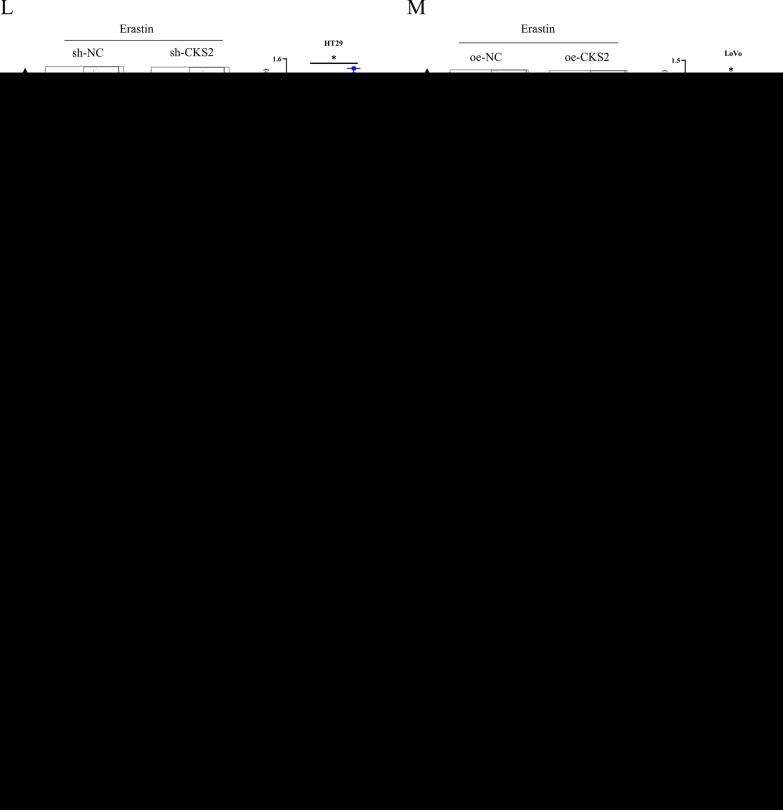
Effects of CKS2 on CC cells ferroptosis. **A** Enriched pathway of CKS2 in bioinformatics analysis; **B** Positive correlation between CKS2 and inhibitory genes in ferroptosis; C: qRT-PCR detected CKS2 expression in cells of each group; **D–E** CCK-8 and clone formation assays measured cell viability and proliferation capacity in each group; **F** TEM observed morphological changes in mitochondria; **G, L, M** BODIPY581/591 staining assessed lipid ROS level in cells; **H, N, O** DCFH-DA staining analyzed cell ROS level; **I** CCK-8 measured IC_50_; **J–K** PI staining detected cell survival; **P** The kit for GSH and GSSG detection determined GSH level; **Q** Glutamate colorimetric kit measured glutamate level; **R–S** Cysteine colorimetric kit assessed cysteine level; **T** Cystine uptake fluorescence kit determined cystine uptake level; **U** The kit for GSH and GSSG detection measured GSH/GSSG ratio. Data are presented as the mean ± SD of three independent experiments. Statistical analysis was conducted by t-test. * means *P* < 0.05

To dig out the role of CKS2 in CC development, this study found that CKS2 was implicated in the ferroptosis signaling pathway in bioinformatics analysis (Fig. 2A). CKS2 was positively correlated with the ferroptosis inhibitory genes (Fig. 2B). To further elucidate the cellular functions, HT29 or LoVo cells were treated with CKS2 knockdown or CKS2 overexpression. qRT-PCR uncovered that knocking down or overexpressing CKS2 considerably reduced or elevated the mRNA levels of CKS2 in HT29 or LoVo cells (Fig. 2C), respectively. CCK-8 and colony formation assays indicated that the viability and proliferative capacity of CC cells with knocked down or overexpressed CKS2 were remarkably lower or higher than those in the control group (Fig. 2D and E). By observing mitochondria by TEM, we unearthed that the membrane density of mitochondria increased and the ridges of mitochondria shrank or disappeared in HT29 cells with CKS2 knocked down. No significant abnormal changes were observed in the mitochondria of the oe-CKS2 group (Fig. 2F). Subsequently, we measured the levels of lipid ROS through BODIPY 581/591 staining, finding that the sh-CKS2 group greatly enhanced the accumulation of lipid ROS. On the contrary, overexpression of CKS2 reduced the lipid ROS level (Fig. 2G). Similar results were observed when we quantified the levels of ROS in cells using DCFH-DA staining (Fig. 2H).

Next, we treated the CC cells from different groups with ferroptosis inducer Erastin at different concentrations (0/5/10/20/40/80 µM). CCK-8 assay uncovered that knocking down CKS2 reduced the IC_50_ value in HT29 cells compared to the control group, while overexpression had the opposite effect (Fig. 2I). Notably, we further treated HT29 (20 µM) or LoVo (28 µM) cells with Erastin after knocking down or overexpressing CKS2. In the presence of Erastin, knocking down CKS2 increased Erastin-induced cell death, while overexpression of CKS2 inhibited it (Fig. 2J and K). Results of BODIPY581/591 staining and DCFH-DA staining demonstrated that CKS2 knockdown increased the accumulation of lipid ROS and cell ROS triggered by Erastin. However, opposite results were observed in cells with CKS2 overexpressed (Fig. 2L - O). GSH, synthesized by glutamate, cysteine, and glycine, is indispensable in ferroptosis as it can eliminate lipid ROS (Chen et al. 2019; Lu 2009). Therefore, we speculated that CKS2-mediated ferroptosis may be related to the GSH metabolic pathway. Corresponding assays by using kits discovered that CKS2 knockdown led to a notable decrease in levels of GSH, cysteine, and cystine uptake in Erastin-treated CC cells, while glutamate levels were considerably increased. Overexpression of CKS2, on the other hand, resulted in the opposite effect on GSH, glutamate, cysteine, and cystine uptake levels (Fig. 2P - T). Furthermore, we resorted to GSH and GSSG assay kits to assess GSH and oxidized glutathione (GSSG), finding that knockdown of CKS2 decreased the Erastin-induced GSH/GSSG ratio, which was elevated by CKS2 overexpression (Fig. 2U). Collectively, overexpression of CKS2 represses the ferroptosis of CC cells, which is linked with enhanced GSH metabolism.

### CKS2 provokes autophagy-mediated GSH metabolism reprogramming in CC cells

**Fig. 3:**
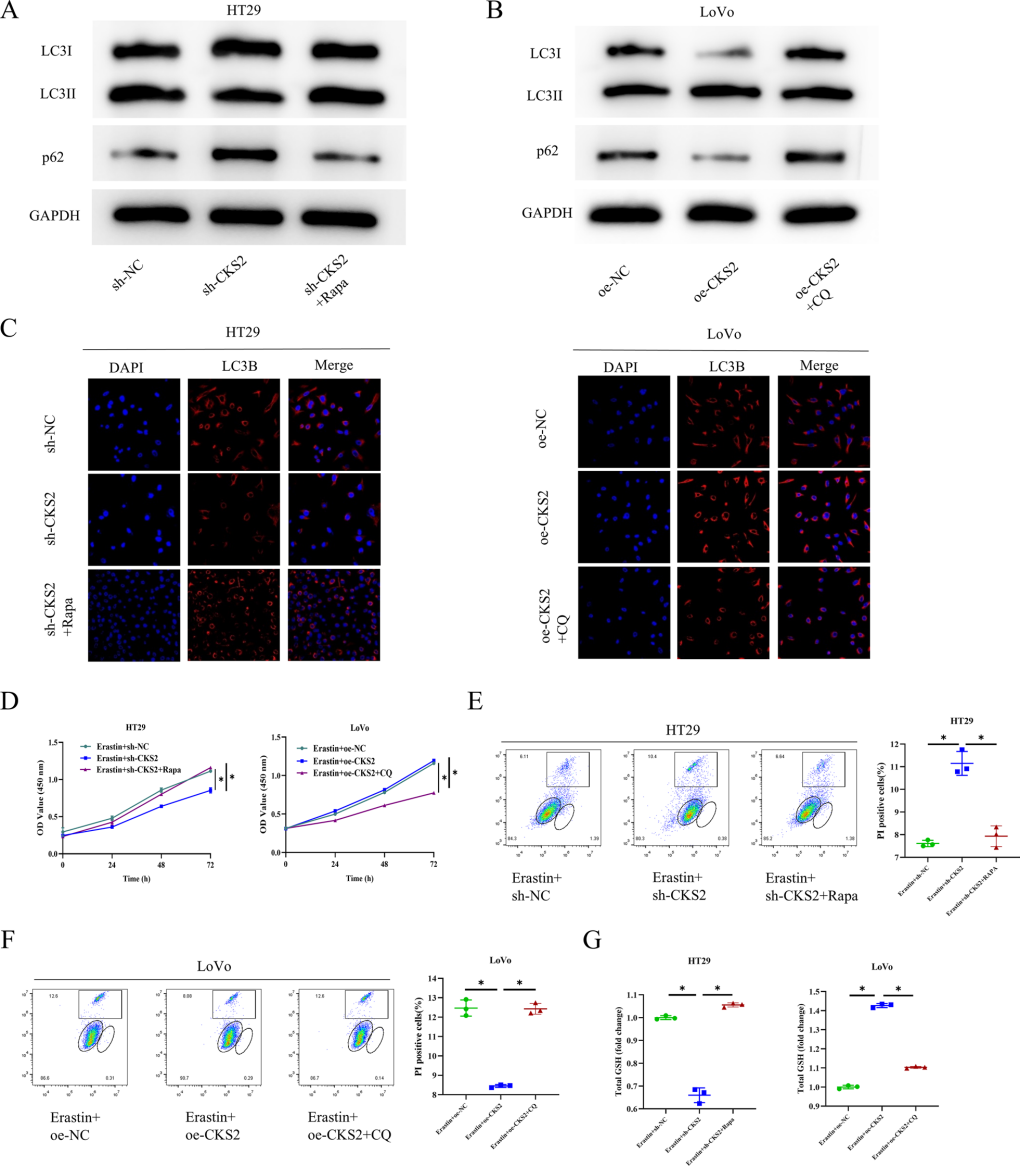

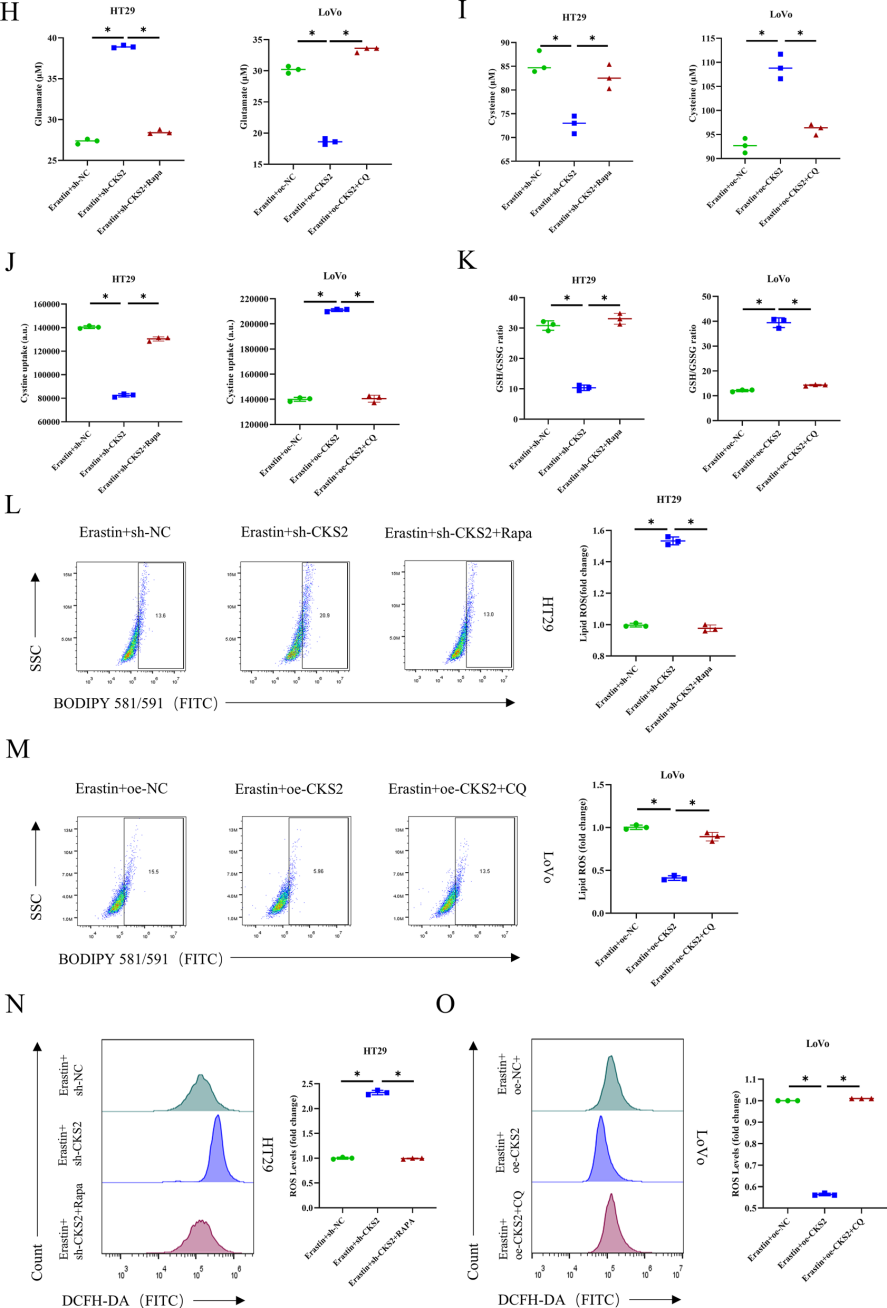
CKS2 mediates GSH metabolism reprogramming through autophagy activation in CC cells **A**, **B **WB measured expression levels of autophagy-related proteins (LC3I, LC3II, and p62); **C** IF detection assessed LC3B expression; **D** CCK-8 assay detected cell viability; **E**, **F** PI staining measured cell survival; **G** The kit for GSH and GSSG detection determined the level of GSH; **H** Glutamate colorimetric kit analyzed the level of glutamate; **I** Cysteine colorimetric kit assessed the level of cysteine; **J** Cystine uptake fluorescence kit determined cystine uptake level; **K** The kit for GSH and GSSG detected GSH/GSSG ratio; **L**, **M** BODIPY581/591 staining tested lipid ROS level; **N**, **O** DCFH-DA staining examined cell ROS level. Data are presented as the mean ± SD of three independent experiments. Statistical analysis was conducted by one-way ANOVA. * means *P*<0.05

Previous studies have supported that differential expression of CKS2 can facilitate the progression of pancreatic cancer by modulating autophagy (Tsang et al. 2020). Moreover, the autophagy can trigger GSH metabolism (Hu et al. 2022; Mukhopadhyay et al. 2021). Therefore, we treated CC cells with autophagy inducer Rapa and autophagy inhibitor CQ to determine whether CKS2-mediated autophagy had relationships with the activity of the GSH metabolism pathway. The cell groups were set as follows: sh-NC, sh-CKS2, sh-CKS2 + Rapa, oe-NC, oe-CKS2, and oe-CKS2 + CQ. First, WB analysis revealed that compared with the sh-NC group, the sh-CKS2 group had the prominently downregulated protein level of LC3II/LC3I while the prominently upregulated protein level of p62. After treatment with Rapa, the expression levels of LC3II/LC3I and p62 were returned to the control level (Fig. 3A). In contrast, in the oe-CKS2 group, the protein level of LC3II/LC3I was considerably increased while the protein level of p62 was considerably reduced, both of which were weakened by CQ treatment (Fig. 3B). Additionally, IF indicated that the expression of LC3B was substantially reduced in the sh-CKS2 group. Rapa treatment attenuated the inhibitory effect conferred by CKS2 knockdown on LC3B protein expression while CQ treatment weakened the impact conferred by CKS2 overexpression on LC3B protein expression (Fig. 3C). Subsequently, we treated the above groups of CC cells with Erastin, obtaining new cell groups as follows: Erastin + sh-NC, Erastin + sh-CKS2, Erastin + sh-CKS2 + Rapa, Erastin + oe-NC, Erastin + oe-CKS2, and Erastin + oe-CKS2 + CQ. As evidenced by CCK-8, the decrease in cell viability of the Erastin + sh-CKS2 group was attenuated after treatment with Rapa. Furthermore, the addition of CQ considerably dampened the cell viability of the Erastin + oe-CKS2 group (Fig. 3D). PI staining indicated that the apoptosis rate of sh-the Erastin + CKS2 group decreased after Rapa treatment (Fig. 3E). Conversely, treatment with CQ elevated the apoptosis rate of the Erastin + oe-CKS2 group (Fig. 3F). Corresponding assay kits showed that the levels of GSH, cysteine, cystine uptake, and GSH/GSSG ratio in the Erastin + sh-CKS2 group were upregulated after treatment with Rapa, while glutamate concentration was reduced. In addition, in CC cells treated with CQ, the levels of GSH, cysteine, cystine uptake, and GSH/GSSG ratio in the Erastin + oe-CKS2 group were decreased after CQ treatment, while glutamate concentration was increased (Fig. 3G - K). Similarly, consistent with the results of CKS2-induced autophagy promotion in GSH metabolism, the upregulation of lipid ROS and cell ROS levels by Erastin + sh-CKS2 was notably weakened after treatment with Rapa. The addition of CQ increased the levels of lipid ROS and cell ROS in the Erastin + oe-CKS2 group (Fig. 3L - O). Taken together, CKS2-induced autophagy mediates the reprogramming of GSH metabolism in CC cells, enhancing cancer resistance in ferroptosis.

### The inhibition of CKS2 enhances ferroptosis by reducing GPX4 expression

**Fig. 4:**
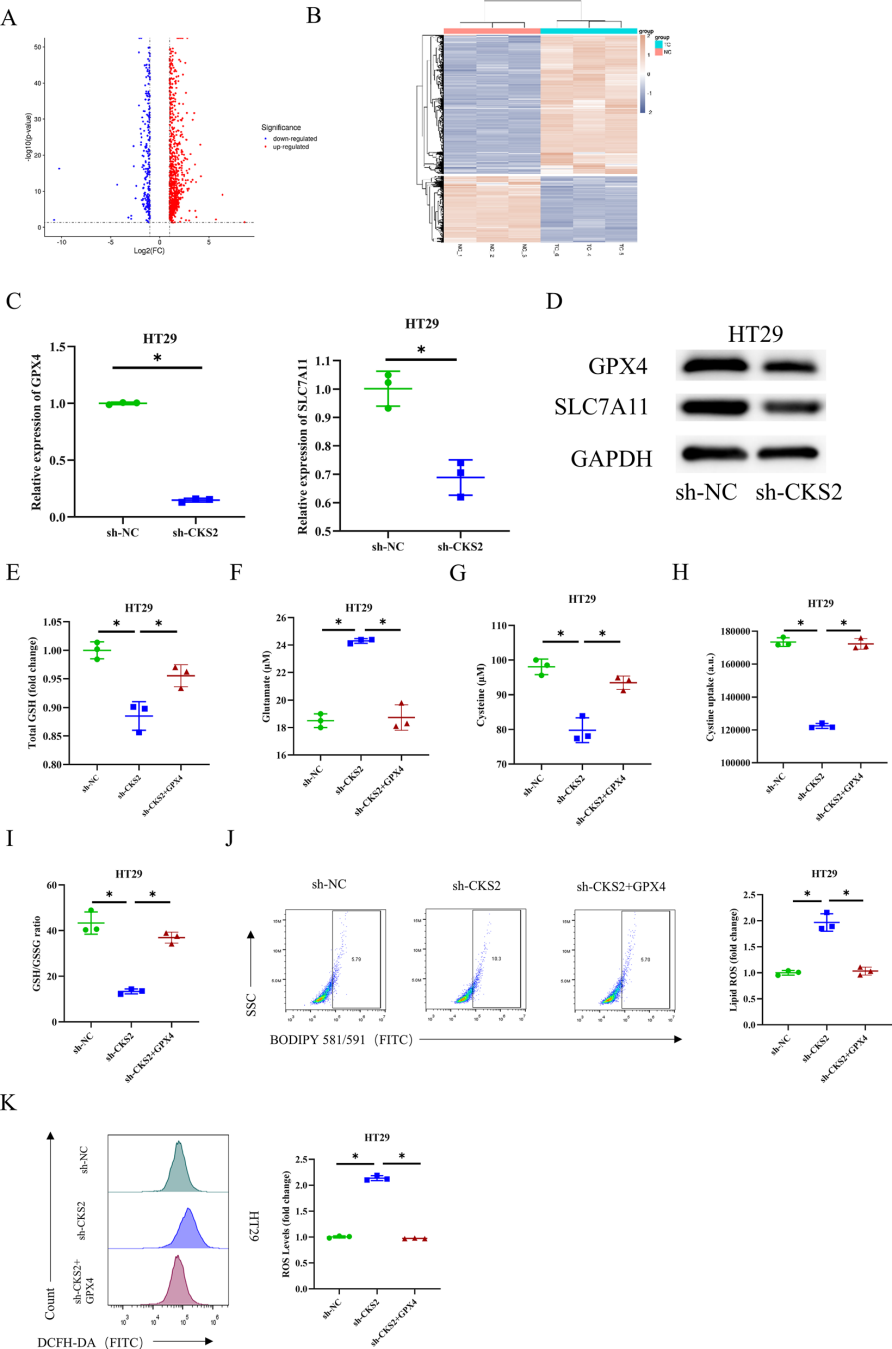
The effect of CKS2 upregulating GPX4 on ferroptosis. **A** Volcano plot of DEGs (DEGs: 1494, upregulated genes: 1011, downregulated genes: 483); **B** Cluster analysis of DEGs, where the horizontal axis represents sample names and clustering results, and the vertical axis represents DEGs and gene clustering results (NC represents sh-NC group, TC represents sh-CKS2 group); **C, D** qRT-PCR and WB detected mRNA and protein expression levels of GPX4 and SLC7A11 in CC cells; **E** The kit for GSH and GSSG determined the level of GSH; **F** Glutamate colorimetric kit assessed the level of glutamate; **G** Cysteine colorimetric kit tested the level of cysteine; **H** Cystine uptake fluorescence kit examined cystine uptake level; **I** The kit for GSH and GSSG detected GSH/GSSG ratio; **J** BODIPY581/591 staining analyzed lipid ROS level; **K** DCFH-DA staining measured cell ROS level. Data are presented as the mean ± SD of three independent experiments. Statistical analysis was conducted by one-way ANOVA. * means *P* < 0.05

We analyzed the genes related to the ferroptosis signaling pathway in CC cells transfected with sh-NC and sh-CKS2 through RNA-seq to further probe into the function of CKS2 in ferroptosis modulation. RNA-seq analysis uncovered a decrease in the activity of GPX4 and SLC7A11 in sh-CKS2 group cells (Fig. 4A and B). We then employed qRT-PCR and WB to assess the mRNA and protein expression of GPX4 and SLC7A11 in CC cells transfected with sh-NC and sh-CKS2, which demonstrated that CKS2 knockdown reduced the mRNA and protein expression of GPX4 and SLC7A11 in HT29 cells compared to the sh-NC group (Fig. 4C and D). Subsequently, we knocked down CKS2 and added recombinant GPX4 protein solution to the culture system. The results of the corresponding assay kits demonstrated that compared to the sh-NC group, the levels of GSH, cysteine, cystine uptake, and GSH/GSSG ratio were substantially decreased in sh-CKS2 cells, while the glutamate level was increased. The inhibitory effect of CKS2 low expression on GSH metabolism, however, was reversed after the addition of recombinant GPX4 protein (Fig. 4E - I). BODIPY staining unearthed that CKS2 knockdown elevated the lipid ROS level in CC cells compared to the control, which was restored to the control level after the addition of recombinant GPX4 protein (Fig. 4J). Similar results were observed when we analyzed the level of cell ROS through DCFH-DA staining (Fig. 4K). Given all results, repressing CKS2 reinforces CC cell ferroptosis by reducing GPX4 expression.

### The repression of CKS2 facilitates sora-induced ferroptosis

**Fig. 5:**
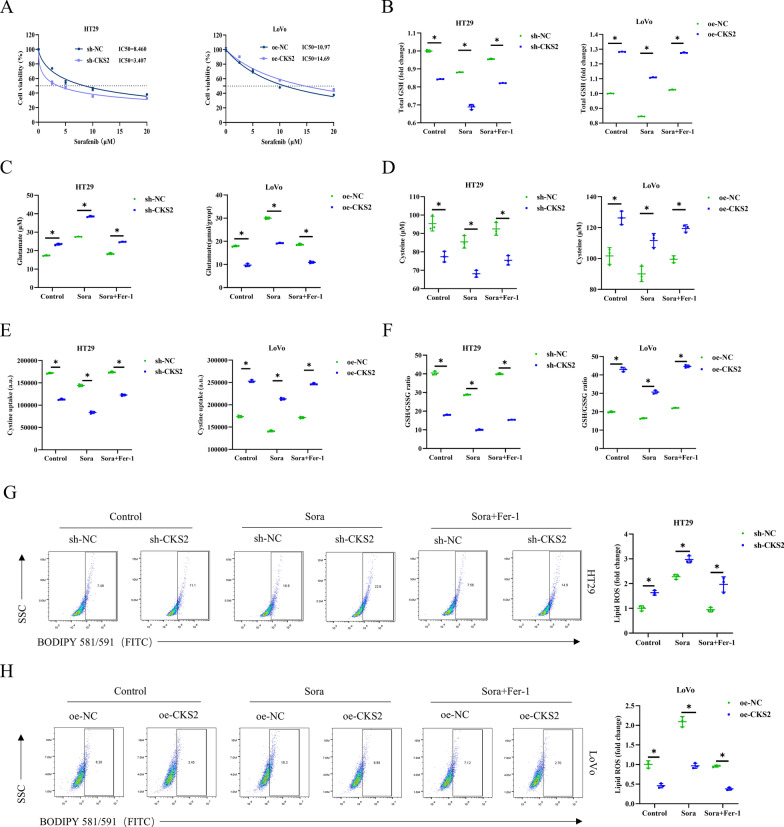
Repression of CKS2 facilitates sora-induced ferroptosis. **A** CCK-8 detected IC_50_; **B** The kit for GSH and GSSG measured the level of GSH; **C** Glutamate colorimetric kit determined the level of glutamate; **D** Cysteine colorimetric kit measured the level of cysteine; **E** Cystine uptake fluorescence kit tested cystine uptake level; **F** The kit for GSH and GSSG detected GSH/GSSG ratio; **G,** **H** BODIPY581/591 staining analyzed lipid ROS level. Data are presented as the mean ± SD of three independent experiments. Statistical analysis was conducted by t-test. * means *P* < 0.05

According to reports, sora can induce ferroptosis in cancer cells through system Xc (Dixon et al. 2014). To dig out the function of CKS2 in sora-induced ferroptosis, we treated HT29 and LoVo cells with gradient concentrations of sora (0/2.5/5/10/20 µM) for 24 h, including cells with CKS2 knocked down or overexpressed and their respective control groups. CCK-8 assay revealed that compared to the sh-NC or oe-NC group, CKS2 knockdown resulted in a decrease in the IC_50_ value of sora in HT29 cells, while CKS2 overexpression increased the IC_50_ value of sora in LoVo cells (Fig. 5A). These data suggested that CKS2 may affect the sensitivity of CC cells to sora treatment. Moreover, we treated CC cells with sora alone or ferroptosis inhibitor (Fer-1) + sora (cell groups: sh-NC, sh-CKS2, sh-NC + Sora, sh-CKS2 + Sora, sh-NC + Sora + Fer-1, sh-CKS2 + Sora + Fer-1, oe-NC, oe-CKS2, oe-NC + Sora, oe-CKS2 + Sora, oe-NC + Sora + Fer-1, and oe-CKS2 + Sora + Fer-1). According to corresponding kits, we observed that knocking down CKS2 led to a more pronounced decrease in sora-induced GSH, cysteine, and cystine levels, while the level of glutamate increased more strikingly. Treatment with Fer-1 slowed down the decrease in levels of GSH, cysteine, and cystine, as well as the increase in glutamate. CKS2 overexpression caused the restoration of decreases in sora-induced GSH, cysteine, and cystine, as well as the increase in glutamate levels, leading them to be close to those of the control group. The addition of Fer-1 further increased the levels of GSH, cysteine, and cystine, while further decreasing the level of glutamate (Fig. 5B - E). At the same time, the GSH and GSSG detection kit demonstrated that knocking down CKS2 leads to a more telling decrease in the sora-induced GSH/GSSG ratio compared to sh-NC + Sora cells, and these effects were attenuated with Fer-1 treatment. However, the opposite results were observed in the CKS2 overexpression condition (Fig. 5F). Finally, we examined the level of lipid ROS through BODIPY581/591 staining. The results indicated that after treatment with sora, sora-induced lipid ROS level in CC cells with CKS2 knocked down was elevated more considerably. The treatment with Fer-1 partially reversed the promoting effect conferred by sh-CKS2 + Sora on lipid ROS (Fig. 5G). Additionally, CKS2 overexpression reversed the increase in lipid ROS level induced by sora. After Fer-1 treatment, lipid ROS in CC cells was further repressed (Fig. 5H). Taken all these findings, CKS2 overexpression suppresses sora-induced ferroptosis in GC cells.

### CKS2 cooperates with sora to induce ferroptosis in nude mice in vivo

**Fig. 6:**
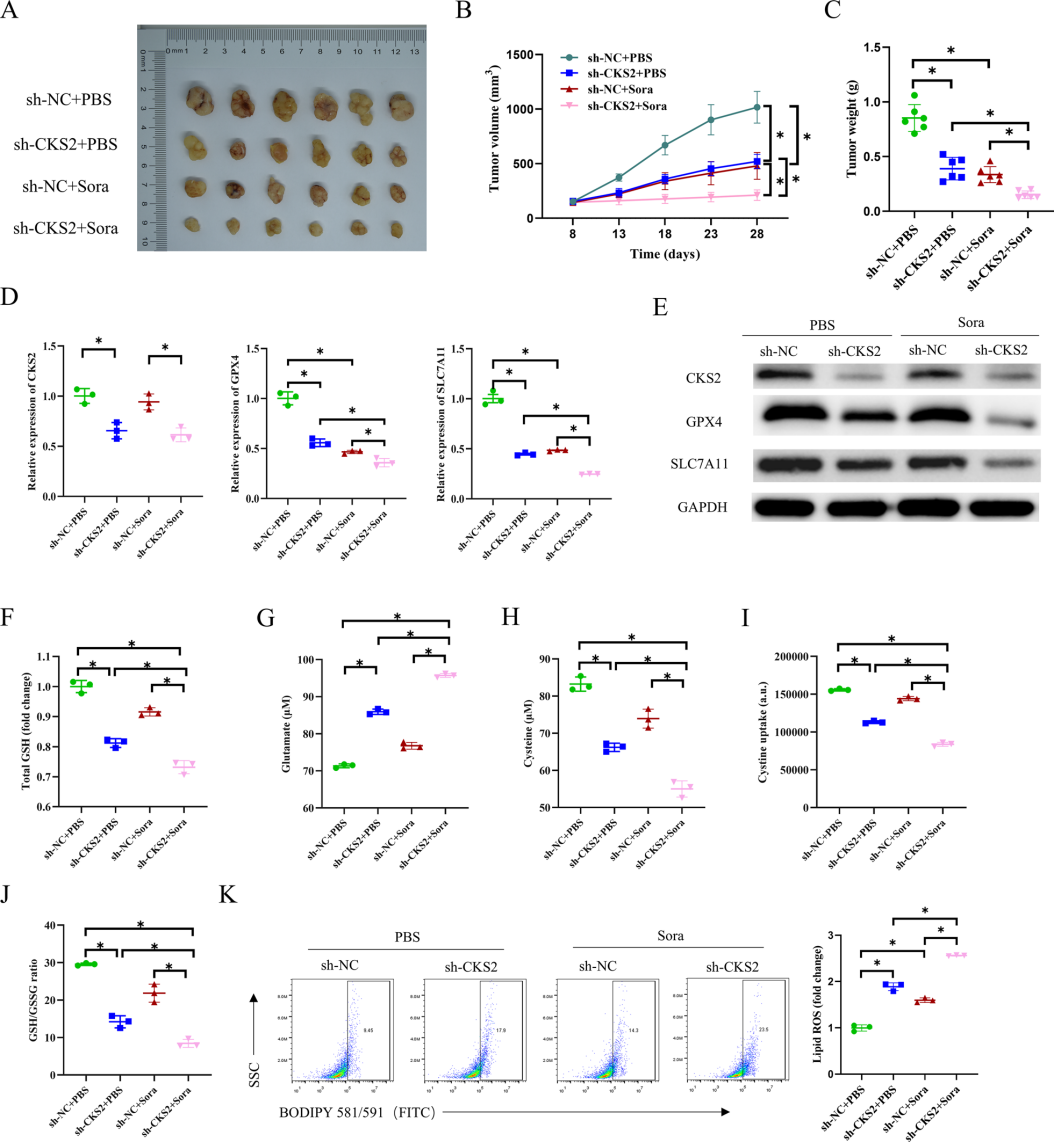
The effect of CKS2 and sora on tumorigenesis in nude mice. **A** Images of tumors in nude mice treated with PBS and sora in sh-NC and sh-CKS2 groups (*n* = 6); **B** Tumor growth curves in sh-NC and sh-CKS2 groups after treatment with PBS and sora (*n* = 6); **C** Tumor weight in sh-NC and sh-CKS2 groups after treatment with PBS and sora (*n* = 6); **D, E** qRT-PCR and WB measured mRNA and protein expression levels of CKS2, GPX4, and SLC7A11 in nude mouse tumors (*n* = 3); **F** The kit for GSH and GSSG determined level of GSH; **G** Glutamate colorimetric kit assessed glutamate level (*n* = 3); **H** Cysteine colorimetric kit detected cysteine level (*n* = 3); **I** Cystine uptake fluorescence kit determined cystine uptake levels (*n* = 3); **J** The kit for GSH and GSSG determined GSH/GSSG ratio (*n* = 3); **K** BODIPY581/591 staining detect lipid ROS level (*n* = 3). Data are presented as the mean ± SD of six/three independent experiments. Statistical analysis was conducted by one-way ANOVA. * means *P* < 0.05

To further evaluate the influence of CKS2 and sora on tumor occurrence in vivo, CC cell lines with CKS2 knockdown and control groups were subcutaneously injected into nude mice. Mice carrying tumors were randomly assigned to receive treatment with PBS or sora. The tumor volume and weight in the CKS2 knockdown group were lower than those in the control group regardless of which treatment. It is worth noting that compared to the sh-NC + PBS and sh-CKS2 + PBS groups, the difference between the CKS2 knockdown group and the control group was more striking after treatment with sora (Fig. 6A - C). Similarly, qRT-PCR and WB measurements demonstrated that the expression of CKS2, GPX4, and SLC7A11 in the sh-CKS2 + PBS group was considerably lower than that in the control group. After sora treatment, CKS2 knockdown further repressed the expression of GPX4 and SLC7A11 (Fig. 6D and E). Subsequently, we examined the levels of GSH, glutamate, cysteine, and cystine uptake using corresponding reagent kits, which uncovered that knocking down CKS2 led to consistent downregulation of GSH, cysteine, and cystine uptake levels as well as upregulation of glutamate level. The degree of upregulation or downregulation was more pronounced in the sora-treated group (Fig. 6F - I). Furthermore, the results of the GSH/GSSG ratio demonstrated that compared to sh-NC + PBS, the sh-CKS2 + PBS group exhibited a decreased GSH/GSSG ratio. Moreover, the GSH/GSSG ratio was considerably reduced in the CKS2 low-expression group treated with sora (Fig. 6J). Finally, the lipid ROS level was assessed using BODIPY581/591 staining, which revealed that the lipid ROS level in the sh-CKS2 + PBS group was remarkably higher than the control group. This increase was more pronounced upon adding sora (Fig. 6K). Collectively, the decrease of CKS2 is synergistic with sora to induce ferroptosis in nude mice.

## Discussion

In this study, we focused on elucidating the mechanism by which CC cells evade ferroptosis, with a specific focus on systematically identifying the CKS2 gene. Previous research has indicated that CKS2 is significantly upregulated in tumors like NSCLC and breast cancer, serving as a potential biomarker for tumor diagnosis or treatment (Wan et al. 2022; Huang et al. 2019). Additionally, CKS2, through activating TGFβ/SMAD signaling, promotes malignant phenotypes and the epithelial-mesenchymal transition process in glioma (Feng et al. 2023). However, the impact of CKS2 on CC progression remains unclear. Our observations revealed elevated levels of CKS2 expression in CC tissues and cells, consistent with bioinformatics database analyses. Moreover, the bioinformatics data indicated a strong correlation between increased CKS2 expression and patient survival rates. CKS2 overexpression was found to suppress the ferroptosis of CC cells, while CKS2 knockdown induced ferroptosis. Based on these findings, we propose that upregulation of CKS2 plays a crucial role in enhancing resistance to ferroptosis in CC.

Distinguished from other forms of cell death, ferroptosis represents a novel non-apoptotic cell demise pathway predominantly observed in tumor cells. Ferroptosis has been reported to impede inflammation and cancer progression at the cellular and organismal levels (Chen et al. 2022a, b). Recent research endeavors have aimed to elucidate the underlying mechanisms of ferroptosis. Increasing evidence suggests that GSH serves as a potent antioxidant crucial for maintaining cellular redox balance, thereby shielding tumor cells from ferroptosis. For instance, actin-like 6 A (ACTL6A) promotes GSH synthesis by elevating the expression of the rate-limiting enzyme GCLC, thereby safeguarding gastric cancer cells from ferroptosis (Yang et al. 2023). In our study, we systematically demonstrated that the induction of ferroptosis by the Erastin ferroptosis inducer led to more pronounced enhancement of ferroptosis and inhibition of GSH metabolism in CC cells with CKS2 knocked down. This finding indicates that CKS2 suppresses ferroptosis in CC cells, specifically through the GSH metabolism pathway. Given the crucial role of GSH in regulating redox balance, certain molecules involved in GSH metabolism, such as GPX4 and SLC7A11, have been identified to impede ferroptosis in cancer cells (Zeng et al. 2022). Wang et al. (Wang et al. 2021a, 2021b). highlighted that SFRS9 can inhibit ferroptosis by increasing GPX4 protein expression, thus promoting CRC progression. Moreover, in CC, the small molecule drug honokiol can restrain cancer cell growth by boosting GPX4 enzyme activity to enhance ferroptosis (Guo et al. 2021). In line with these investigations, RNA-seq analysis revealed a reduction in the activity of GPX4 and SLC7A11 in the sh-CKS2 group. Silencing CKS2 significantly suppressed the expression of GPX4 and SLC7A11, leading to ferroptosis in CC cells. Moreover, the promotion of ferroptosis in CC cells due to CKS2 knockdown was attenuated when cells were treated with a recombinant GPX4 protein solution. This observation provides a deeper understanding of the role of ferroptosis in cancer, presenting potential avenues for diagnosis and therapeutic intervention in CC.

Previous research has clarified that CKS2 is capable of suppressing the autophagy process in LUAD (Chen et al. 2022a, b). Our research has revealed a contrasting role of CKS2 in inducing autophagy and orchestrating a reprogramming of GSH metabolism in CC cells. This discrepancy suggests that the function of CKS2 may exhibit variability across different cancer types, and autophagy could potentially influence ferroptosis in CC cells by modulating GSH metabolism. Autophagy, as a cellular pathway, represents a double-edged sword that can hinder tumor initiation while also accelerating tumor progression (Russell and Guan 2022). While a certain association between ferroptosis and autophagy has been established (Liu et al. 2020), there is no unanimous consensus regarding the interplay between these two processes. Several studies suggest a connection between ferroptosis and the activation of autophagy. For instance, Cdc25A upregulates ErbB2 via the PKM2-pH3T11-H3K9Ac pathway, thereby shielding CC cells from autophagy-induced ferroptosis (Wang et al. 2021a, 2021b). Conversely, numerous pieces of evidence also indicate that autophagy can suppress ferroptosis. For instance, PNO1 enhances GSH biosynthesis by promoting autophagy, consequently diminishing the susceptibility of hepatocellular carcinoma (HCC) cells to ferroptosis (Hu et al. 2022). The relationship between ferroptosis and autophagy remains an unresolved issue. In this study, by assessing cell viability, survival, lipid content, and cellular levels of ROS following treatment with the autophagy inducer Rapa and the autophagy inhibitor CQ, we have inferred that CKS2 boosts GSH metabolism and inhibits ferroptosis in CC cells by stimulating autophagy.

Sora is an FDA-approved multi-kinase inhibitor drug used in the treatment of various cancers, including renal cell carcinoma and HCC (Rini et al. 2020; Yau et al. 2022). Recently, a growing body of evidence has shown that sora induces ferroptosis to kill cancer cells. For example, SLC27A5 is downregulated in HCC, enhancing sora-induced ferroptosis by inhibiting the NRF2/GSR pathway in cancer cells, presenting a potential therapeutic approach to overcome sora resistance (Xu et al. 2023). In our study, we observed a parallel effect of sora in CC cells. Sora treatment substantially increased the levels of reactive oxygen species (ROS) in CC cells while reducing the level of glutathione (GSH). Furthermore, our investigation revealed that silencing CKS2 augmented the impact of sora treatment on ferroptosis both in vitro and in vivo. These findings propose that CKS2 could serve as a promising therapeutic target for overcoming sora resistance in CC cells. However, this hypothesis needs validation in patients, highlighting the need for further research in the future.

In summary, our study indicates that CKS2-driven autophagy promotes resistance to ferroptosis in CC through GSH metabolic reprogramming, both in vitro and in vivo. To the best of our knowledge, our study is the first to propose this concept. Additionally, we demonstrated that inhibiting CKS2 enhances ferroptosis and boosts sora-induced ferroptosis by reducing GPX4 expression. Despite these advancements, our work has some limitations. Firstly, the involvement of CKS2 in other forms of ferroptosis induced by different agents, such as RSL3, remains unexplored and warrants further investigation for validation. Secondly, clinical trials are necessary to provide additional evidence supporting our research outcomes. In conclusion, our findings have the potential to offer new perspectives on early CC diagnosis and rational drug targets for the treatment of CC patients.

## Data Availability

No datasets were generated or analysed during the current study.

## References

[CR1] Chen Y, Zhu G, Liu Y, Wu Q, Zhang X, Bian Z, et al. O-GlcNAcylated c-Jun antagonizes ferroptosis via inhibiting GSH synthesis in liver cancer. Cell Signal. 2019;63:109384. 31394193 10.1016/j.cellsig.2019.109384

[CR2] Chen X, Kang R, Kroemer G, Tang D. Broadening horizons: the role of ferroptosis in cancer. Nat Reviews Clin Oncol. 2021;18:280–96. 10.1038/s41571-020-00462-0 33514910

[CR3] Chen L, Hu K, Liu Y, Liu L, Tang J, Qin X. Knockdown of replication protein A 3 induces protective autophagy and enhances cisplatin sensitivity in lung adenocarcinoma by inhibiting AKT/mTOR signaling via binding to cyclin-dependent kinases regulatory subunit 2. Drug Dev Res. 2022a;83:1589–99. 35903032 10.1002/ddr.21978

[CR4] Chen H, Wang C, Liu Z, He X, Tang W, He L, et al. Ferroptosis and its multifaceted role in cancer: mechanisms and therapeutic approach. Antioxid (Basel). 2022;11:1504. 10.3390/antiox11081504 PMC9405274 36009223

[CR5] Chen Y, Lin B, Yang S, Huang J. IRF1 suppresses colon cancer proliferation by reducing SPI1-mediated transcriptional activation of GPX4 and promoting ferroptosis. Exp Cell Res. 2023;431:113733. 37517591 10.1016/j.yexcr.2023.113733

[CR6] Dixon SJ, Patel DN, Welsch M, Skouta R, Lee ED, Hayano M, et al. Pharmacological inhibition of cystine-glutamate exchange induces endoplasmic reticulum stress and ferroptosis. Elife. 2014;3:e02523. 24844246 10.7554/eLife.02523 PMC4054777

[CR7] Feng F, Zhao Z, Cai X, Heng X, Ma X. Cyclin-dependent kinase subunit2 (CKS2) promotes malignant phenotypes and epithelial-mesenchymal transition-like process in glioma by activating TGFbeta/SMAD signaling. Cancer Med. 2023;12:5889–907. 36284444 10.1002/cam4.5381 PMC10028050

[CR8] Guo J, Xu B, Han Q, Zhou H, Xia Y, Gong C, et al. Ferroptosis: a novel anti-tumor action for cisplatin. Cancer Res Treat. 2018;50:445–60. 28494534 10.4143/crt.2016.572 PMC5912137

[CR9] Guo C, Liu P, Deng G, Han Y, Chen Y, Cai C, et al. Honokiol induces ferroptosis in colon cancer cells by regulating GPX4 activity. Am J Cancer Res. 2021;11:3039–54. 34249443 PMC8263670

[CR10] Han L, Yan Y, Fan M, Gao S, Zhang L, Xiong X, et al. Pt3R5G inhibits colon cancer cell proliferation through inducing ferroptosis by down-regulating SLC7A11. Life Sci. 2022;306:120859. 35931199 10.1016/j.lfs.2022.120859

[CR11] Hu X, He Y, Han Z, Liu W, Liu D, Zhang X, et al. PNO1 inhibits autophagy-mediated ferroptosis by GSH metabolic reprogramming in hepatocellular carcinoma. Cell Death Dis. 2022;13:1010. 36446769 10.1038/s41419-022-05448-7 PMC9709074

[CR12] Huang N, Wu Z, Hong H, Wang X, Yang F, Li H. Overexpression of CKS2 is associated with a poor prognosis and promotes cell proliferation and invasion in breast cancer. Mol Med Rep. 2019;19:4761–9. 30957190 10.3892/mmr.2019.10134 PMC6522797

[CR13] Jiang X, Stockwell BR, Conrad M. Ferroptosis: mechanisms, biology and role in disease. Nat Rev Mol Cell Biol. 2021;22:266–82. 33495651 10.1038/s41580-020-00324-8 PMC8142022

[CR14] Li B, Wei S, Yang L, Peng X, Ma Y, Wu B, et al. CISD2 promotes resistance to Sorafenib-Induced ferroptosis by regulating Autophagy in Hepatocellular Carcinoma. Front Oncol. 2021;11:657723. 34485112 10.3389/fonc.2021.657723 PMC8415543

[CR15] Liberal V, Martinsson-Ahlzen HS, Liberal J, Spruck CH, Widschwendter M, McGowan CH, et al. Cyclin-dependent kinase subunit (Cks) 1 or Cks2 overexpression overrides the DNA damage response barrier triggered by activated oncoproteins. Proc Natl Acad Sci U S A. 2012;109:2754–9. 21697511 10.1073/pnas.1102434108 PMC3286935

[CR16] Liu J, Long S, Wang H, Liu N, Zhang C, Zhang L, et al. Blocking AMPK/ULK1-dependent autophagy promoted apoptosis and suppressed colon cancer growth. Cancer Cell Int. 2019;19:336. 31871431 10.1186/s12935-019-1054-0 PMC6911288

[CR17] Liu J, Kuang F, Kroemer G, Klionsky DJ, Kang R, Tang D. Autophagy-dependent ferroptosis: machinery and regulation. Cell Chem Biol. 2020;27:420–35. 32160513 10.1016/j.chembiol.2020.02.005 PMC7166192

[CR18] Lu SC. Regulation of glutathione synthesis. Mol Aspects Med. 2009;30:42–59. 18601945 10.1016/j.mam.2008.05.005 PMC2704241

[CR19] Ma Q, Chang Z, Wang W, Wang B. Rapamycin-mediated mTOR inhibition reverses drug resistance to adriamycin in Colon cancer cells. Hepatogastroenterology. 2015;62:880–6. 26902021

[CR20] Martinsson-Ahlzen HS, Liberal V, Grunenfelder B, Chaves SR, Spruck CH, Reed SI. Cyclin-dependent kinase-associated proteins Cks1 and Cks2 are essential during early embryogenesis and for cell cycle progression in somatic cells. Mol Cell Biol. 2008;28:5698–709. 18625720 10.1128/MCB.01833-07 PMC2546922

[CR21] Mukhopadhyay S, Biancur DE, Parker SJ, Yamamoto K, Banh RS, Paulo JA, et al. Autophagy is required for proper cysteine homeostasis in pancreatic cancer through regulation of SLC7A11. Proc Natl Acad Sci U S A. 2021;118:e2021475118. 33531365 10.1073/pnas.2021475118 PMC8017731

[CR22] Qin L, Luo X, Qin X, Huang H, Zhang L, Chen S, et al. Comprehensive expression profiling and molecular basis of cdc28 protein kinase regulatory subunit 2 in cervical cancer. Int J Genomics. 2022;2022:6084549. 35935749 10.1155/2022/6084549 PMC9352497

[CR23] Rini BI, Pal SK, Escudier BJ, Atkins MB, Hutson TE, Porta C, et al. Tivozanib versus Sorafenib in patients with advanced renal cell carcinoma (TIVO-3): a phase 3, multicentre, randomised, controlled, open-label study. Lancet Oncol. 2020;21:95–104. 31810797 10.1016/S1470-2045(19)30735-1

[CR24] Rosander E, Holm T, Sjovall A, Hjern F, Weibull CE, Nordenvall C. Preoperative multidisciplinary team assessment is associated with improved survival in patients with locally advanced colon cancer; a nationwide cohort study in 3157 patients. Eur J Surg Oncol. 2021;47:2398–404. 34112562 10.1016/j.ejso.2021.05.008

[CR25] Russell RC, Guan KL. The multifaceted role of autophagy in cancer. EMBO J. 2022;41:e110031. 35535466 10.15252/embj.2021110031 PMC9251852

[CR26] Sung H, Ferlay J, Siegel RL, Laversanne M, Soerjomataram I, Jemal A, et al. Global Cancer statistics 2020: GLOBOCAN estimates of incidence and Mortality Worldwide for 36 cancers in 185 countries. CA Cancer J Clin. 2021;71:209–49. 33538338 10.3322/caac.21660

[CR27] Tang D, Chen X, Kang R, Kroemer G. Ferroptosis: molecular mechanisms and health implications. Cell Res. 2021;31:107–25. 33268902 10.1038/s41422-020-00441-1 PMC8026611

[CR28] Tsang YH, Wang Y, Kong K, Grzeskowiak C, Zagorodna O, Dogruluk T, et al. Differential expression of MAGEA6 toggles autophagy to promote pancreatic cancer progression. Elife. 2020;9:e48963. 32270762 10.7554/eLife.48963 PMC7164953

[CR29] Wan Z, Wang L, Yang D, Li P, Liu Q, Wang B. CKS2 promotes the growth in Non-small-cell Lung Cancer by Downregulating cyclin-dependent kinase inhibitor. Pathobiology. 2022;89:13–22. 34333494 10.1159/000517755

[CR30] Wang R, Xing R, Su Q, Yin H, Wu D, Lv C, et al. Knockdown of SFRS9 inhibits progression of Colorectal Cancer through triggering ferroptosis mediated by GPX4 reduction. Front Oncol. 2021a;11:683589. 34336668 10.3389/fonc.2021.683589 PMC8322952

[CR31] Wang C, Zeng J, Li LJ, Xue M, He SL. Cdc25A inhibits autophagy-mediated ferroptosis by upregulating ErbB2 through PKM2 dephosphorylation in cervical cancer cells. Cell Death Dis. 2021b;12:1055. 34743185 10.1038/s41419-021-04342-y PMC8572225

[CR32] Wu J, Liu C, Wang T, Liu H, Wei B. Deubiquitinase inhibitor PR-619 potentiates colon cancer immunotherapy by inducing ferroptosis. Immunology. 2023;170:439–51. 37526037 10.1111/imm.13683

[CR33] Xu FL, Wu XH, Chen C, Wang K, Huang LY, Xia J, et al. SLC27A5 promotes sorafenib-induced ferroptosis in hepatocellular carcinoma by downregulating glutathione reductase. Cell Death Dis. 2023;14:22. 36635256 10.1038/s41419-023-05558-w PMC9837139

[CR34] Yang Z, Zou S, Zhang Y, Zhang J, Zhang P, Xiao L, et al. ACTL6A protects gastric cancer cells against ferroptosis through induction of glutathione synthesis. Nat Commun. 2023;14:4193. 37443154 10.1038/s41467-023-39901-8 PMC10345109

[CR35] Yau T, Park JW, Finn RS, Cheng AL, Mathurin P, Edeline J, et al. Nivolumab versus Sorafenib in advanced hepatocellular carcinoma (CheckMate 459): a randomised, multicentre, open-label, phase 3 trial. Lancet Oncol. 2022;23:77–90. 34914889 10.1016/S1470-2045(21)00604-5

[CR36] Ye S, Xu M, Zhu T, Chen J, Shi S, Jiang H, et al. Cytoglobin promotes sensitivity to ferroptosis by regulating p53-YAP1 axis in colon cancer cells. J Cell Mol Med. 2021;25:3300–11. 33611811 10.1111/jcmm.16400 PMC8034452

[CR37] Yu MH, Luo Y, Qin SL, Wang ZS, Mu YF, Zhong M. Up-regulated CKS2 promotes tumor progression and predicts a poor prognosis in human colorectal cancer. Am J Cancer Res. 2015;5:2708–18. 26609478 PMC4633900

[CR38] Yuan Y, Xiao WW, Xie WH, Cai PQ, Wang QX, Chang H, et al. Neoadjuvant chemoradiotherapy for patients with unresectable radically locally advanced colon cancer: a potential improvement to overall survival and decrease to multivisceral resection. BMC Cancer. 2021;21:179. 33607964 10.1186/s12885-021-07894-6 PMC7893883

[CR39] Zeng C, Lin J, Zhang K, Ou H, Shen K, Liu Q, et al. SHARPIN promotes cell proliferation of cholangiocarcinoma and inhibits ferroptosis via p53/SLC7A11/GPX4 signaling. Cancer Sci. 2022;113:3766–75. 35968603 10.1111/cas.15531 PMC9633309

[CR40] Zeng XY, Qiu XZ, Wu JN, Liang SM, Huang JA, Liu SQ. Interaction mechanisms between autophagy and ferroptosis: potential role in colorectal cancer. World J Gastrointest Oncol. 2023;15:1135–48. 37546557 10.4251/wjgo.v15.i7.1135 PMC10401467

[CR41] Zhang XM, Wang J, Liu ZL, Liu H, Cheng YF, Wang T. LINC00657/miR-26a-5p/CKS2 ceRNA network promotes the growth of esophageal cancer cells via the MDM2/p53/Bcl2/Bax pathway. Biosci Rep. 2020;40:BSR20200525. 32426838 10.1042/BSR20200525 PMC7268253

[CR42] Zheng X, Wang Q, Zhou Y, Zhang D, Geng Y, Hu W, et al. N-acetyltransferase 10 promotes colon cancer progression by inhibiting ferroptosis through N4-acetylation and stabilization of ferroptosis suppressor protein 1 (FSP1) mRNA. Cancer Commun (Lond). 2022;42:1347–66. 36209353 10.1002/cac2.12363 PMC9759759

