# Correction: CKS2 induces autophagy-mediated glutathione metabolic reprogramming to facilitate ferroptosis resistance in colon cancer

**DOI:** 10.1186/s10020-024-01041-0

**Published:** 2025-01-08

**Authors:** Leilei Yang, Chengfeng Fang, Jiaju Han, Yufeng Ren, Zaiping Yang, Lingyan Shen, Dinghai Luo, Ruili Zhang, Yan Chen, Shenkang Zhou

**Affiliations:** 1 Department of Gastrointestinal Surgery, Taizhou Hospital, Wenzhou Medical University, No.105 Westgate Street, Linhai, 317000 China; 2 Key Laboratory of Minimally Invasive Techniques & Rapid Rehabilitation of Digestive System Tumor of Zhejiang Province, Linhai, 317000 China; 3 Department of Anaesthesia, Taizhou Hospital, Wenzhou Medical University, Linhai, 317000 China; 4 Department of Gastroenterology, Taizhou Hospital, Wenzhou Medical University, Linhai, 317000 China; 5 Department of Family-Oriented Wards, Taizhou Hospital, Wenzhou Medical University, No.105 Westgate Street, Linhai, 317000 China

**Correction** **:** **Molecular Medicine (2024) 30:219** 10.1186/s10020-024-00979-5

In the original publication of this article (Yang et al. 2024), the author “Chen Yan” should have been denoted as a corresponding author.

The original article has been corrected.
